# Crucial Role of the CB3-Region of Collagen IV in PARF-Induced Acute Rheumatic Fever

**DOI:** 10.1371/journal.pone.0004666

**Published:** 2009-03-02

**Authors:** Katrin Dinkla, Susanne R. Talay, Matthias Mörgelin, Rikki M. A. Graham, Manfred Rohde, D. Patric Nitsche-Schmitz, Gursharan S. Chhatwal

**Affiliations:** 1 Department of Microbial Pathogenesis, Helmholtz Centre for Infection Research, Braunschweig, Germany; 2 Department of Clinical Sciences, BMC B14, Lund University, Lund, Sweden; University of Giessen Lung Center, Germany

## Abstract

Acute rheumatic fever (ARF) and rheumatic heart disease are serious autoimmune sequelae to infections with *Streptococcus pyogenes*. Streptococcal M-proteins have been implicated in ARF pathogenesis. Their interaction with collagen type IV (CIV) is a triggering step that induces generation of collagen-specific auto-antibodies. Electron microscopy of the protein complex between M-protein type 3 (M3-protein) and CIV identified two prominent binding sites of which one is situated in the CB3-region of CIV. In a radioactive binding assay, M3-protein expressing *S. pyogenes* and *S. gordonii* bound the CB3-fragment. Detailed analysis of the interactions by surface plasmon resonance measurements and site directed mutagenesis revealed high affinity interactions with dissociation constants in the nanomolar range that depend on the recently described collagen binding motif of streptococcal M-proteins. Because of its role in the induction of disease-related collagen autoimmunity the motif is referred to as “peptide associated with rheumatic fever” (PARF). Both, sera of mice immunized with M3-protein as well as sera from patients with ARF contained anti-CB3 auto-antibodies, indicating their contribution to ARF pathogenesis. The identification of the CB3-region as a binding partner for PARF directs the further approaches to understand the unusual autoimmune pathogenesis of PARF-dependent ARF and forms a molecular basis for a diagnostic test that detects rheumatogenic streptococci.

## Introduction

Acute rheumatic fever (ARF) can occur as a sequela to inadequately treated infection with *Streptococcus pyogenes* (group A streptococcus (GAS)), and it is one of the most serious outcomes of streptococcal disease [1]. ARF, which often develops into rheumatic heart disease (RHD), remains a major cause of cardiovascular disease that is affecting the young, particularly in developing countries [1], [2]. Recent data estimate that more than 15 million people suffer from RHD, more than 0.5 million acquire ARF each year, and about 0.25 million deaths annually are directly attributable to either ARF or RHD [2].

Although the exact pathogenesis of ARF remains elusive, it is known to be the result of autoimmune responses triggered by streptococcal infection [1], [3]. Type IV collagen (CIV) is a major constituent of endothelial cell basement membranes, and is a factor which is involved in a series of autoimmune syndromes [4]. Certain streptococcal strains are able to bind collagen and this interaction is important for virulence [5]–[7]. Six genetically distinct alpha-chains of CIV exist, which assemble into hetero-trimers of different compositions. These molecules consist of a triple-helical domain that is flanked by non-collagenous domains, the N-terminal 7S domain and the C-terminal globular domain, referred to as NC1 (for schematic representation see [8]). Both non-collagenous domains are involved in the formation of hexagonal networks that are the typical assembly of CIV in the basement membrane. CIV binds to cells by interacting with α_1_β_1_ and α_2_β_1_ integrins via a region that is located approximately 100 nm from the N-terminus and that is known as cyanogen bromide fragment 3 (CB3) [9], [10].

A major virulence factor of *S. pyogenes* is the M-protein. This surface protein exists in more than 100 serotypes which is the consequence of a high sequence variability in the N-terminal part of the protein. The rheumatogenicity of *S. pyogenes* strains has been shown to correlate with certain M serotypes, suggesting that the M-protein plays a key role in the pathogenesis of ARF [11]. One such rheumatogenic type is the M3 serotype. Immunization of mice with M3-protein leads to the formation of CIV auto-antibodies, which are also found in the sera of patients with ARF or RHD [6] and our group has shown previously that collagen-binding M-proteins like the M3-protein bind and aggregate CIV via an octa-peptide motif that is referred to as PARF (peptide associated with rheumatic fever) [5]. The observation that the collagen autoimmunity that is caused by PARF does not depend on molecular mimicry [5] motivated further investigations on the molecular details of the interaction between M-proteins and CIV. The herein described insights will help us to elucidate the induction of ARF-related collagen autoimmunity, which to date is poorly understood.

## Results

### Characterization of the interaction between CIV and M3-protein

**Figure 1:**
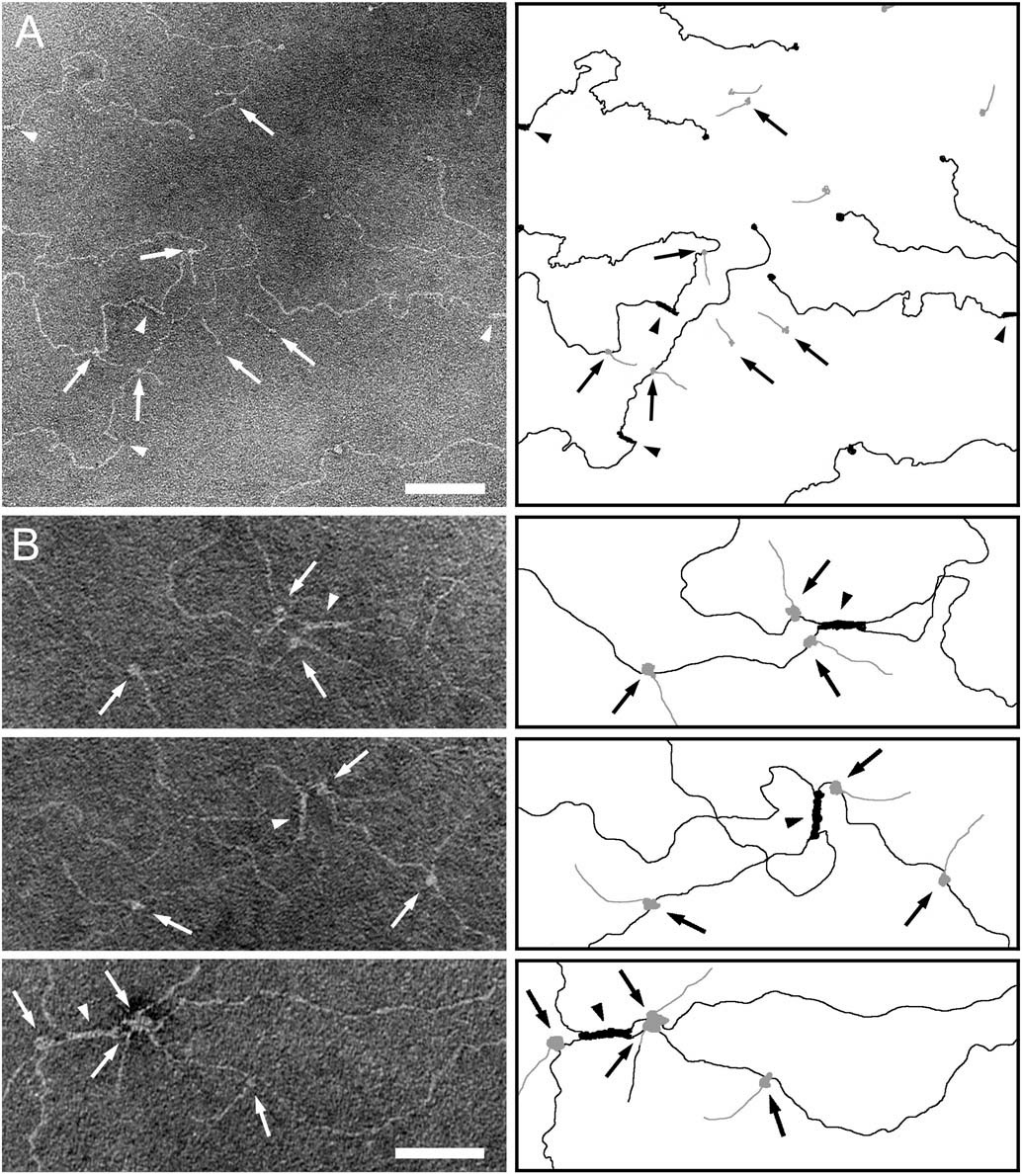
Electron microscopy of M3-CIV complexes. Micrographies (*left panel*) and corresponding cartoons (*right panel*) show complexes that consist of M3-protein and CIV. Block arrows highlight M3-protein (depicted in gray in the cartoons), both free and bound to CIV (depicted in black in the cartoons). Arrow heads point out the 7S regions of CIV, while line arrows points out the globular heads of the CIV molecules. The bars represent 100 nm in the representative overviev (*A*) and 50 nm in the panel of selected complexes (*B*).

**Figure 2:**
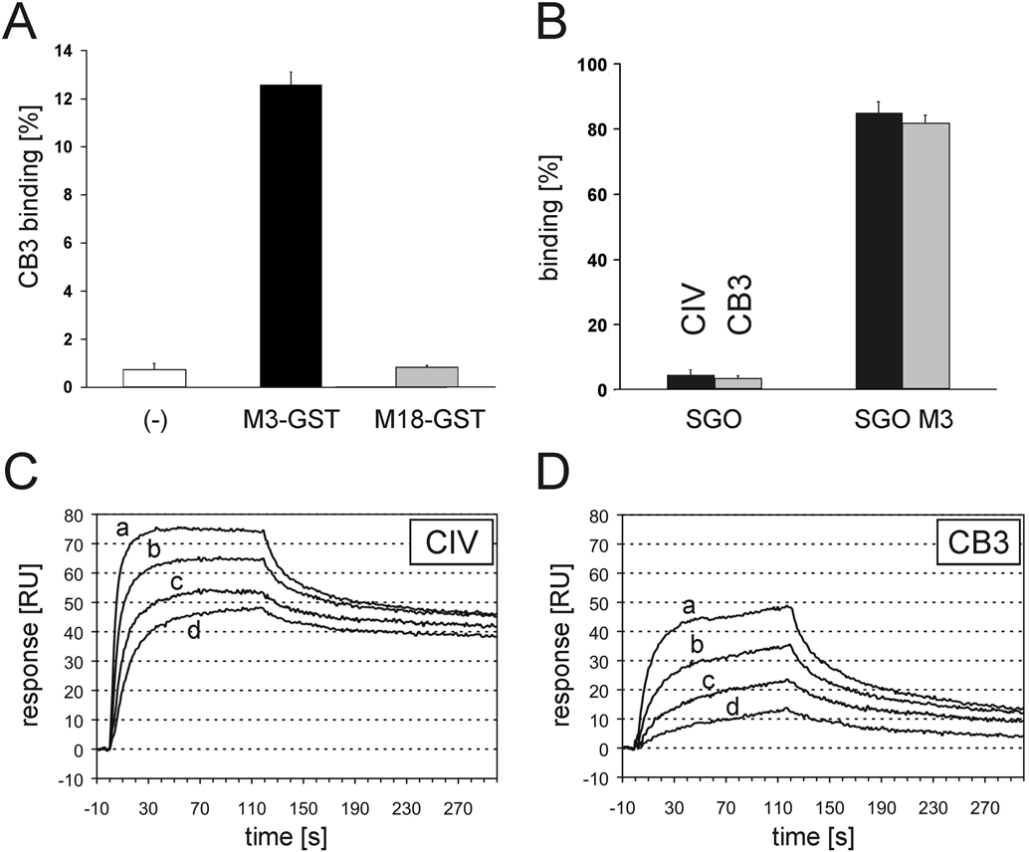
CB3 binding by M3-protein. (*A*) Interaction with ^125^I-CB3 was measured in a pull-down assay with, *M3-GST* (*black bar*), or *M18-GST* (*grey bar*) protein coupled to glutathion sepharose beads. A control sample with glutathion separose alone (−) was included (*white bar*). (*B*) Binding of ^125^I-CIV (black bars) or ^125^I-CB3 (*grey bars*) to *S. gordonii* that heterologously expressed M3 protein on its surface (*SGO M3*) and that of the wild type control (*SGO*) was expressed as a percentage of total radioactivity input. Error bars in *A* and *B* represent the standard deviation of the triplicate measurements. (*C* and *D*) Surface plasmon resonance measurements of the interaction between M3-protein and immobilized CIV (*C*) or CB3 (*D*), respectively. Injection of M3-protein at different concentrations (a: 50 µg/ml, b: 25 µg/ml, c: 12.5 µg/ml, d: 6.25 µg/ml) started at t = 0 s and stopped at t = 120 s. The response is expressed in response units (RU).

Examination of complexes between CIV and M3-protein by means of rotary shadowing electron microscopy gave insights into the molecular basis of the interaction. Consistent with previous observations on the complex of FOG and collagen I [7] the M-protein appeared as a thread-like structure with a globular end that is formed by the N-terminal GST-tag. As shown in Figure 1, the N-terminal end of the M3-protein (*arrows*) forms contacts with the CIV-molecule. M3-protein bound to two prominent sites on the CIV-molecule, 20 nm and 100 nm distant from the 7S region (*arrow heads*). The latter site corresponds to the position of the CB3 region of CIV. In a pull-down assay glutathion sepharose beads were loaded with GST-tagged M-proteins type M3 and M18, respectively. While M3-protein bound ^125^I-labeled CB3, M18-protein produced radioactivity counts at the level of the GST-loaded glutathion sepharose that was used as a negative control (Fig. 2A). Surface bound M3-protein on SGO M3 also bound high amounts of both CIV and CB3 (≥80% of the ^125^I-labeled ligand) (Fig. 2B), as seen in comparison with the wild type *S. gordonii* that showed no binding to either full length CIV or the CB3-fragment. When M3-protein was subjected to surface plasmon resonance analysis using either full length CIV or the CB3-fragment of CIV as an immobilized ligand it showed concentration dependent binding to both CIV (Fig. 2C) and CB3 (Fig. 2D), with apparent dissociation constants of K_d_ = 5×10^−9^ M and K_d_ = 6×10^−8^ M, respectively. Taken together the results demonstrate a direct and specific interaction between M3-protein and CIV and identify a binding site in the CB3-region of CIV that exhibits a considerable affinity. Expression of M3-protein on its surface is sufficient to transform the *S. gordonii* strain into a bacterium with a high binding capacity for CIV and CB3.

### PARF is the binding motif for CB3

**Figure 3:**
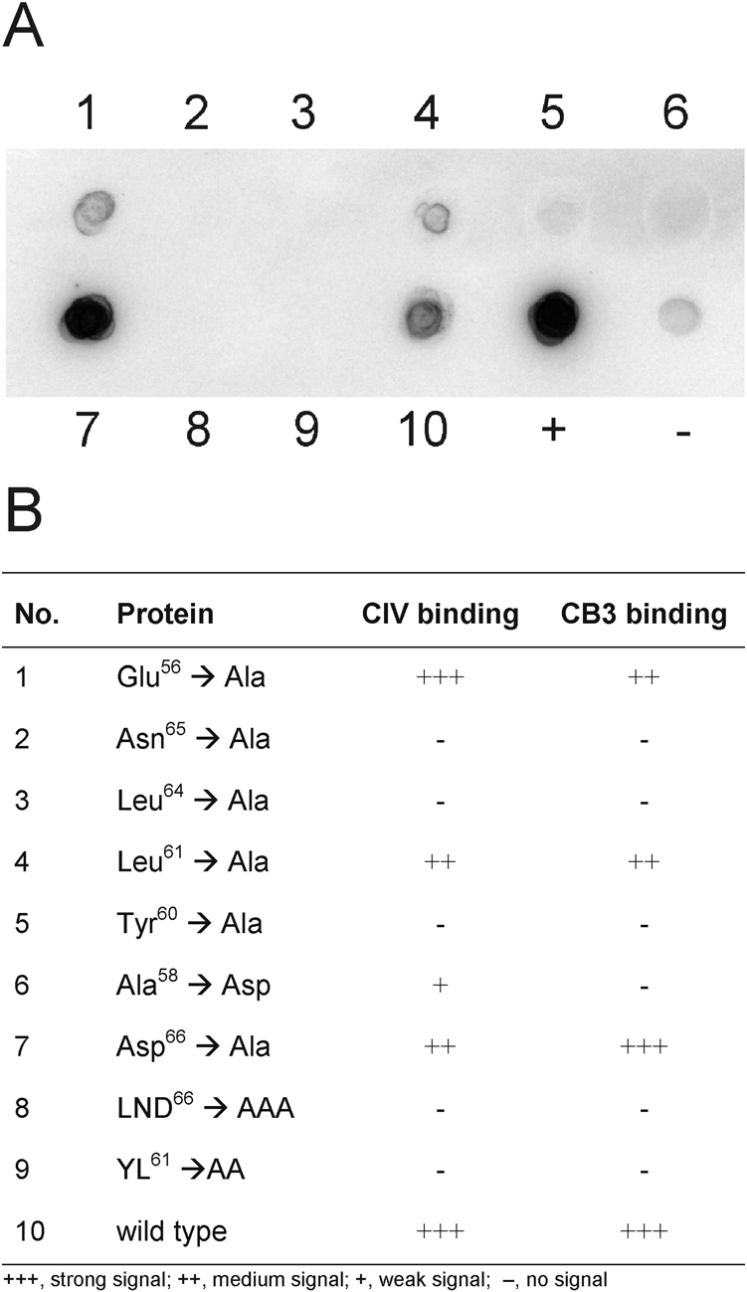
Binding of PARF mutants to CB3. (*A*) Dot blot experiment with ^125^I-CB3 as the soluble ligand. Dots 1–9 are the immobilized mutants of M protein FOG (for description see table in *B*); dot 10 is recombinant wild type protein. Recombinant M3- (+) and M18-proteins (−) were used as positive and negative control, respectively. The table in *B* allows to compare the experiment with previous data on full length collagen IV[5].

Recently, we have identified the consensus motif that is common to all collagen binding streptococcal M-proteins and could demonstrate that it has a crucial role in the pathogenesis of rheumatic fever [5]. Its consensus sequence AXYLZZLN is designated PARF (peptide associated with rheumatic fever). To test its role in binding to CB3, dot-blot experiments with wild type M-protein and a set of variants with mutations in PARF [5] were conducted. The results, which are summarized in figure 3, widely paralleled previous ones on full length CIV [5], mutants with a defective PARF (with substitutions in Ala^58^, Tyr^60^, Leu^61^, Leu^64^, Asn^65^, LND^66^ or YL^61^) did not bind to CB3, whereas mutations outside of PARF (Glu^56^, Asp^66^) did not affect CB3 binding (Fig. 3). Hence, an intact PARF-motif in the M-protein is essential for efficient binding to CB3.

### M3-protein induces autoimmunity against CB3

**Figure 4:**
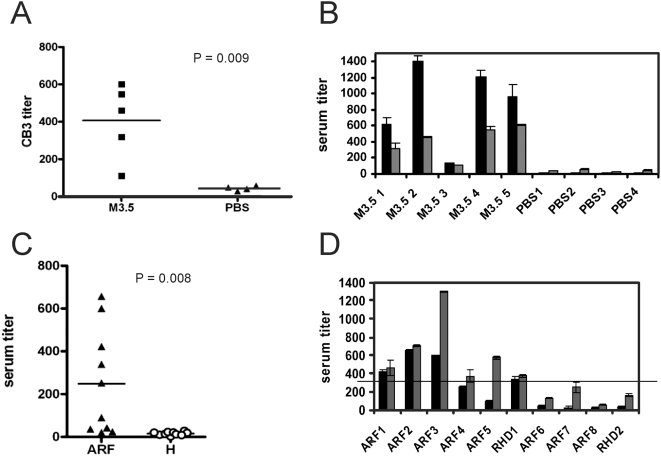
CIV and CB3 specific serum responses. (*A*) Serum Ig response for CB3 in M3.5 immunized mice. Reactivity was determined for sera from PBS-immunized (PBS) or M3-protein-immunized (M3.5) mice. The anti-CIV (*black bars*) and anti-CB3 titers (*grey bars*) measured in individual mice immunized with M3.5 (*M3.5 #*) or PBS (PBS #) are compared in (*B*). (*C*) Anti-CB3 titer of individual patient sera. Titers are shown for each individual serum collected from ARF/RHD patients (triangles), or healthy individuals (circles). The significance of the differences measured in *A* and *C* was examined using the t-test. (*D*) The six sera of patients with CB3 autoimmunity (black bars) showed also reactivity against the collagen binding part of M3-protein (M3.5) (grey bars). The horizontal line indicates the cut-off titer for M3.5 positive sera. Error bars represent the standard deviation of the triplicate measurements in *B* an *D*.

Immunization of mice with either full length M3-protein [6] or the type specific collagen binding N-terminal part of M3-protein (M3.5) [5] leads to the induction of CIV auto-immunity. To examine the contribution of the CB3-region in the elicitation of the immune response, titers of CB3-reactive antibodies in the sera of mice immunized with M3.5 were measured and found to be significantly elevated as compared to buffer controls (P = 0.0085) (Fig. 4A). All murine sera were reactive against CIV and had antibodies against the CB3-fragment (Fig. 4B). Analysis of human sera from clinical cases of collagen-associated ARF/RHD revealed elevated titers against CB3 in six of ten human sera (Fig. 4C) giving evidence for the presence of self-epitopes in this region of CIV. Interestingly these six sera also had positive titers against the collagen binding N-terminal M3-fragment (M3.5) whereas vice versa sera with a negative titer against CB3 did not possess M3.5-antibodies (Fig. 4D). Taken together the results demonstrate that immunization of mice with the collagen binding type specific N-terminal part of the M3-protein (M3.5) generates immune response against the CB3-region - its binding site in collagen IV. A similar immune response was found in serum of patients, which indicates a role of this response in the pathogenesis of ARF. The results strongly suggest that binding of M3-protein induces structural changes in the CB3-region rendering this region of CIV auto-antigenic.

## Discussion

ARF and subsequent RHD are serious autoimmune sequelae of *S. pyogenes* infection, and previous studies have demonstrated that the rheumatogenic potential of *S. pyogenes* strains is associated with certain M-types [11]. M3 is one such rheumatogenic serotype and it has been shown to interact with CIV, leading to the production of auto-antibodies against collagen. High titers of CIV-reactive antibodies in sera from patients with ARF are evidence for the clinical relevance of CIV-autoimmunity [6]. Molecular mimicry has been implicated in the induction of myosin auto-immunity [12]. However, experimental data that indicate molecular mimicry of the rheumatogenic serotype M3 are missing. The observation, that CIV auto-antibodies which are induced in mice by injection of collagen binding M-proteins do not cross-react with the respective M-protein, points towards an influence of the M-protein on the presentation of collagen as a self-antigen rather than molecular mimicry [5], [6]. The recent finding, that induction of such an autoimmune response by collagen binding M-proteins depends on the presence of the PARF motif, suggests a critical role of collagen binding in this process [5].

Characterization of the interaction between CIV and M3-protein by electron microscopy at the molecular level identified two distinct binding sites on the CIV-molecule, one situated in the CB3-region, which is known to bare the motif for interactions with the A-domain of α1- and α2 integrin [9], [10]. The cyanogen bromide cleavage product CB3 is a disulfide-bonded heterotrimer of two α1(IV) and two α2(IV) fragments. It retains a triple-helical structure that is necessary for integrin binding [9], [10]. The structural requirements and influences of the interaction between CB3 and streptococcal M-protein are not yet described but promise to shed more light on its role in streptococcal pathogenesis. The observation that M3-protein binds to the integrin binding fragment of CIV suggests that it interferes with CIV-integrin interactions, modulates them or exploits them for the benefit of the bacterium. Experiments with the collagen-binding M-protein of group G streptococci, FOG, indicate that the PARF-motif is involved in the CB3-interaction (Fig. 3), which underscores the importance of this motif in the interaction between streptococcal M-proteins and CIV [5]. The lack of molecular mimicry together with the data obtained on PARF-containing M-proteins suggest that the herein described interaction evokes autoantigenic properties of the CB3-region. Mice immunized with the N-terminal CIV-binding fragment of M3 (M3.5) developed CB3 auto-antibodies, indicating that the region bares a potent self-epitope. Anti-CB3-titers in the sera of ARF patients demonstrate a clinical relevance of this self-epitope. In this context, it is noteworthy that another CIV-dependent autoimmune disease, the Goodpasture Syndrome, is caused by exposure of cryptic epitopes to the immune system [4], which fosters the notion of similarities in the pathogenesis of collagen-dependent ARF. Hence, further examination of the structural influences that are exerted by the PARF-motif, when it is bound to CB3-region, promise insights that are fundamental for the understanding of collagen-dependent ARF.

Moreover, the identification of a smaller fragment of collagen as a receptor for PARF is a considerable step ahead in the development of a specific diagnostic test for rheumatogenic streptococcal strains. Since only about 5% of all *S. pyogenes* strains have the potential to cause ARF, their detection would identify the relatively low proportion of patients – mostly children – that need an intensive treatment to prevent the disease; a strategy with the potential to improve the efficiency of prevention programs for endemic areas, until an effective vaccine against *S. pyogenes* is available.

## Methods

### Bacterial strains

Streptococci were routinely grown in Todd-Hewitt broth supplemented with 5% Yeast extract (THY) or Tryptic Soy Broth (TSB), with antibiotics where appropriate (3 µg/ml erythromycin (Ery), 500 µg/ml kanamycin (Kan)) at 37°C without agitation. *E.coli* strains used for overexpression of recombinant proteins were grown at 37°C with agitation in Luria broth with appropriate antibiotics (100 µg/ml ampicillin (Amp)).

### Rotary metal shadowing transmission electron microscopy of protein complexes

Complexes between M3-protein and collagen IV were analyzed by mica sandwich squeezing/rotary shadowing and transmission electron microscopy. Samples (final concentration of 10 µg/ml) were dialyzed overnight against 0.2 M ammonium hydrogen carbonate, pH 7.9, in a microdialysis apparatus (5–50 µl, Biowerk). They were subsequently mixed with an equal volume of 80% glycerol and squeezed between two freshly cleaved mica pieces for 5 minutes. Specimens were dried at high vacuum for 1–2 h and rotary shadowed at a 9° angle with 2 nm platinum/carbon, followed by coating with 10 nm carbon at a 90° angle. Replicas were floated off onto distilled water and picked up on 400 mesh copper grids and then examined in a Jeol JEM 1230 electron microscope operated at 60 kV accelerating voltage and at calibrated magnifications. Images were recorded with a Gatan Multiscan 791 CCD camera.

### Collagen binding assays

Human placental full length CIV (Sigma-Aldrich) or the CB3-fragment that was prepared as described previously[13], were labeled with ^125^I by the chloramine T method [14]. Approximately 14 ng of ^125^I-CIV or ^125^I-CB3 was added to 250 µl of bacterial suspension and incubated at room temperature for 45 min. The cells were harvested by centrifugation, the supernatant carefully aspirated and pellet-associated radioactivity measured using an automated Wallac gamma counter (Perkin Elmer, Jugesheim, Germany). All measurements were carried out in triplicates. The results were expressed as the percentage of input radioactivity.

### Cloning and expression of recombinant proteins

For generation of GST–M3-protein, *emm3* was amplified from the chromosomal DNA of the M3 strain via PCR using the following primers: M3-1: 5′GCA GAC AGT AGG ATC CGA TGC AGG AGT G 3′ (*Bam* HI restriction site); M3-3: 5′ GAG CAG CTT CAA CGT CGA CTT TTG CTT GGC 3′ (*Sal* I restriction site). Standard cloning techniques were used to generate constructs in the pGEX6P-1 vector system (Amersham Biosciences Europe GmbH, Freiburg, Germany). Proteins were expressed, and purified under native conditions following the manufacturer's protocol.

### Radioactive pull down experiments

Pull down experiments were performed by mixing 50 µg soluble purified recombinant M3-GST or M18-GST fusion protein with 0.5 µg soluble radiolabeled cyanogen bromide fragment CB3 of CIV in a total volume of 70 µl PBS. The mixture was incubated for 45 min at 22°C prior to addition of 100 µl of a 50% glutathion sepharose solution in PBS. The samples were then incubated for 30 min at 22°C, then beads were washed 3× with 1 ml PBST, precipitated, and the amount of sepharose bound CB3/M-protein complex was determined by measuring the radioactivity of the pellet in a Wallac gamma counter (Perkin Elmer, Jugesheim, Germany).

### Surface plasmon resonance measurements

Protein interactions were studied in a BIAcore 2000 system (BIAcore AB) using 10 mM HEPES, 100 mM NaCl, pH = 7.4 as running buffer. A CM5 sensor chip was activated by a 4 min injection of 0.05 M N-hydroxysuccinimid, 0.2 M N-ethyl-N-(3-dime-thylaminopropyl)-carbodiimide hydrochloride in water. Collagen IV from human placenta (Sigma, 1 mg/ml in 0.1 M NaAc) or cyanogens bromide fragment CB3 of collagen type IV was diluted 1∶25 in 10 mM NaAc (pH = 5.2). Injection of 3 µl at a flow rate of 5 µl/min led to immobilization of 400 to 550 response units (RU) of collagen IV. Residual reactive groups were inactivated by a 6 min injection of 1 M ethanolamine, 0.1 M NaHCO_3_, 0.5 M NaCl, 5 mM EDTA, pH = 8.0. Surface regeneration was achieved by injection of two 30 s pulses of 0.2% SDS in water. The BIAevaluation 3.0 software was used for further analysis of the data. Shown curves represent the difference between the signal of the collagen-coupled surface and of a deactivated control surface devoid of protein. They were further corrected by subtraction of the curve that was obtained after injection of buffer alone. Buffer injection led to responses less than 5 RU.

### Construction of recombinant S. gordonii that expresses M3-protein

M3-protein was heterologously expressed on the surface of *S. gordonii* GP1221 using the host integration system described by Oggioni and Pozzi [15]. The *emm3* gene was amplified via PCR using the primers M3-1 and M3-3 which are described above, cloned into plasmid vector pSMB103 to give functional in frame fusion with the signal sequence and the membrane anchor coding regions of *emm6* gene, and inserted via gene replacement as gene cassette into a defined chromosomal locus of the *S. gordonii* chromosome, resulting in surface expression of a functional full length M3-protein. Surface expression of M3-protein was examined by using antibodies specifically recognizing the N-terminal part of the M3-protein.

### Ligand overlay assay

The ligand overlay assay was performed by spotting the purified wild type protein and mutants of the collagen binding M-protein FOG (5 µg) [5] on nitrocellulose. The membrane was blocked for 1 h in PBS containing 5% skim milk followed by a 1-h incubation with radiolabeled CB3-fragment (200,000 cpm) in PBST. After five washing steps in PBST, filters were dried and placed on radiographic films (Eastman Kodak Co.) for auto-radiography.

### Immunization of mice and detection of antibodies

Pathogen free 6–7 week old female C3H/He mice were immunized intra-peritoneally with 100 µg of a recombinant, purified N-terminal fragment of M3-protein (M3.5) (n = 5) or PBS, suspended in 100 µl Freund's incomplete adjuvant per dose at day 1, 7 and 14. At day 21, serum samples of each group were collected, pooled, and tested by ELISA. All studies were approved by the appropriate authorities (*Niedersächsisches Landesamt für Verbraucherschutz und Lebensmittelsicherheit*).

A capture ELISA was used to determine the presence of anti-collagen antibodies in sera. Microtiter plates (Greiner, Frickenhausen, Germany) were coated overnight at 4°C with anti-human CIV rabbit serum (Progen, Heidelberg, Germany) diluted 1∶100 in coating buffer, and blocked with 2% bovine serumalbumin (BSA). Sera (diluted 1∶50 to 1∶50,000 in serial dilutions in PBS) were added to wells and incubated over night at 4°C. After washing, a 1∶1000 dilution of horseradish peroxidase (HRP) conjugated goat anti-mouse Ig (Jackson Laboratories) was added and incubated for 1 h. Antibody binding was detected using 2,2-azino-di-[3-ethylbenzthiazoline sulfonate] diammonium salt (ABTS tablets, Boehringer, Mannheim, Germany) as substrate. The absorbance was determined at 405 nm in triplicates.

To determine anti-CB3 antibody titers, 96-well plates (Greiner, Frickenhausen, Germany) were coated overnight at 4°C with CB3 (2 µg/ml in PBS) and blocked with 2% bovine serum albumin (BSA) in phosphate buffered saline (PBS) for 1 h at room temperature. Sera (diluted 1∶50 to 1∶50,000 in serial dilutions in PBS) were added to wells and incubated over night at 4°C. Antibody binding was measured as described above.

### Patient sera

Sera from ARF patients and healthy controls have been described in [5]. To determine anti-CB3 or anti-M3.5 titer CB3 or M3.5-protein were immobilized overnight at 4°C followed by blocking with 1% bovine serum albumin in PBST. Wells were washed before human sera diluted 1∶50 to 1∶50,000 in serial dilutions in PBS were added to wells and incubated over night at 4°C. After washing, a 1∶5000 dilution of horseradish peroxidase (HRP) conjugated rabbit anti-human Ig (Sigma) was added and incubated for 1 h at 37°C. Antibody binding was detected using 2,2-azino-di-[3-ethylbenzthiazoline sulfonate] diammonium salt (ABTS tablets, Boehringer, Mannheim, Germany) as substrate. The absorbance was determined at 405 nm in triplicates.

To determine the presence of anti-collagen antibodies in sera a capture ELISA was used. Microtiter plates (Greiner, Frickenhausen, Germany) were coated overnight at 4°C with anti-human CIV rabbit serum (Progen, Heidelberg, Germany). After washing with PBST 1% bovine serum albumin in PBS was added to the wells and incubated for 1.5 h at 37°C. Human sera (diluted 1∶50 to 1∶50,000 in serial dilutions in PBS) were added to wells and incubated over night at 4°C. Antibody binding was measured as described above. The highest titer found in symptom free donors was defined as cut-off [5].
